# Supplementary material for: Optimizing core collections for genetic studies: a worldwide flax germplasm case study
Source: Front Plant Sci. 2025 Oct 21;16:1675815. doi: 10.3389/fpls.2025.1675815 (PMC12599843; doi:10.3389/fpls.2025.1675815)
Supplement: Supplementary file 1 [file DataSheet1.docx]

**Supplementary Figure S1:** Tracy-Widom test statistics according to the principal components

**
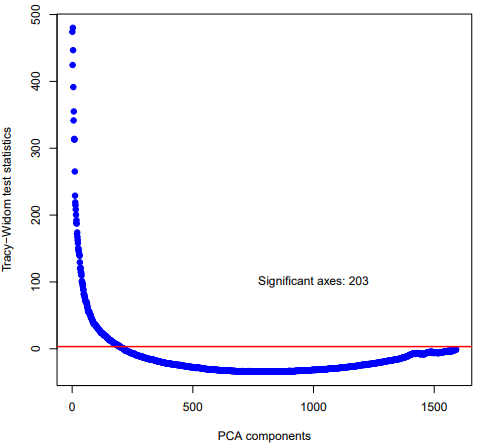
**

**Supplementary Figure S2:**

a) Genomic control inflation factor distribution computed for the whole collection after GWAS analysis

b) Genomic control inflation factor distribution computed for the 200 core-collections collection after GWAS analysis.


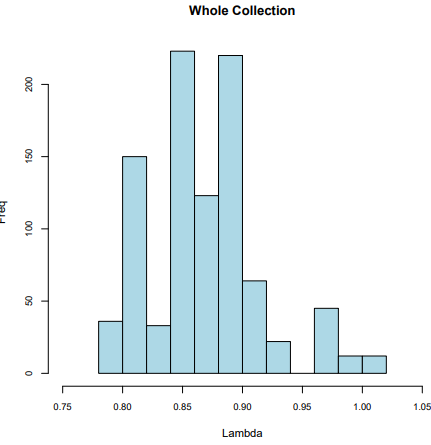


**A**

**
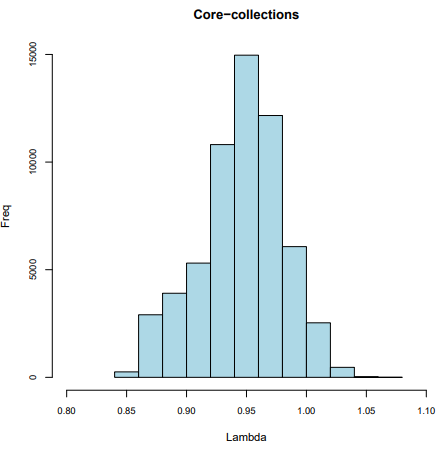
**

**B**

**Supplementary Figure S3**

Number of detected QTLs based on core-collection creation methods. For each method tested, 10 core-collections were created. QTLs were simulated from the whole collection.

**
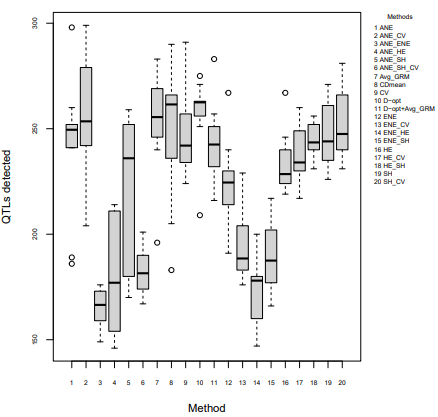
**

**Supplementary Figure S4**

Distribution of the diversity and representativeness criteria and their pairwise Pearson’s correlation coefficient computed for the 200 core-collections created.

**
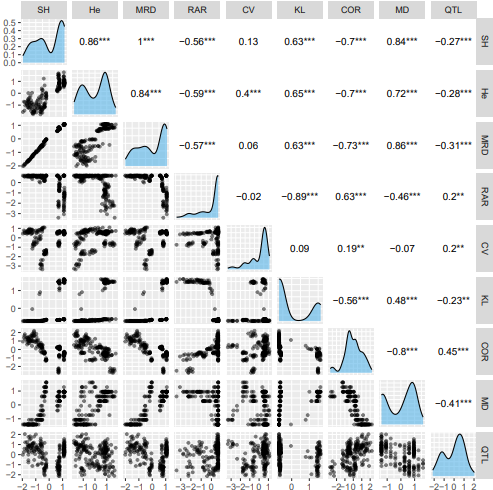
**
